# Just a stopgap for ‘real’ sports? Experiences with digital sport and exercise activities during the COVID-19 pandemic

**DOI:** 10.1007/s12662-023-00881-y

**Published:** 2023-04-25

**Authors:** Michael Mutz, Malte Jetzke, Arne Göring

**Affiliations:** 1 Institute of Sport Science, Justus-Liebig-University Giessen, Kugelberg 62, 35394 Giessen, Germany; 2 Institute of Sport Science, Westfaelische Wilhelms-University Muenster, Muenster, Germany; 3 Center for University Sports, Georg-August-University Goettingen, Goettingen, Germany

**Keywords:** Fitness centers, Digitalization, Physical activity, Online fitness, Membership retention

## Abstract

The coronavirus disease 2019 (COVID-19) pandemic has led to an increase of digitally supported home-based sport and exercise activities. This paper asks how these digital sport and exercise (DSE) offers are experienced by consumers and compares the experiential value of DSE activities with similar on-site, offline sport and exercise (OSE) activities. The paper is based on cross-sectional online survey data from active members of two university sports centers in Germany. Findings reveal that a relative strength of DSE compared to similar OSE offers is the higher degree of autonomy experienced by users. However, this comes at the expense of the social, physical, affective, competence, and motivational experience. DSE courses are somewhat better rated by self-disciplined and healthy individuals, but lower rated by participants with a high motor competence. Overall, data lend support to the notion that DSE was just a stopgap for many during the lockdowns. For becoming a real alternative to on-site sports, DSE offers should be developed further in such a way that emotional and social experiences are enhanced.

**Supplementary Information:**

The online version of this article (10.1007/s12662-023-00881-y) contains supplementary material, which is available to authorized users.

## Introduction

The coronavirus disease 2019 (COVID-19) pandemic has disrupted social activities and public life in Germany and elsewhere. In the first wave of COVID-19 infections, peaking in April 2020, German authorities reacted with a strict lockdown of non-essential public infrastructure. These containment policies included the closing of leisure and sports infrastructure. In a second wave of rising incidence values starting in November 2020, a second ‘lockdown light’ was put in force, where sports infrastructure was closed again. This lockdown lasted until May 2021.

Organized sport is a crucial part of the German sporting landscape, so that the closing of sports facilities deprived millions from opportunities to exercise and play sport and, as a consequence, led to declining levels of sports activity during the lockdowns (Mutz & Gerke, 2021). The German Olympic Sports Confederation counts 27 million memberships (German Olympic Sports Confederation, 2020) and 10 million Germans hold a membership in a commercial fitness club (German Association of Fitness Studios, 2020). Voluntary sports clubs and commercial gyms constantly had to adapt to political regulations and develop strategies to prevent membership losses. The lockdowns faced a substantial share of clubs and gyms with existential fears (Feiler & Breuer, 2021). First estimations—although always to be interpreted with caution—indicate a loss of sport club members of roughly 3.5% or 1,000,000 memberships (Burrmann, Sielschott, & Braun, 2022; Deutscher Bundestag, 2021; Thieme & Wallrodt, 2021).

A key strategy to prevent membership losses was the development of digital sport and exercise (DSE) offers. Many of the clubs and gyms reacted to the COVID-19 pandemic with increased social media activities and the development of DSE courses (Kehl, Strobl, Tittlbach, & Loss, 2021). Global surveys indicate that professionals in the health and fitness sector regard ‘online fitness’ as the most important fitness trend during the pandemic (Thompson, 2021). In Germany and the United Kingdom, for instance, every fifth adult participated in DSE activities during lockdowns (Mutz, Müller, & Reimers, 2021; Sport England, 2020). It can be assumed that a substantial proportion of the population came in contact with DSE activities for the first time. In both countries, however, the use of DSE declined during summer 2020, when sports facilities reopened. Against this background, the question arises how DSE activities are experienced—in their own right as well as in comparison with offline sport and exercise (OSE) activities.

Consumers are often conceptualized as rational actors who allocate scarce resources to products and activities aiming to obtain a desired benefit. In case of leisure activities, the benefit may be better expressed in intrinsic, experiential terms (e.g., wellbeing) instead of extrinsic terms (e.g., monetary rewards). The concept of “experiential rationality” (Schulze, 1992) suggests that consumers try to maximize experiential and hedonic returns through consumption decisions. Leisure activities can have various experiential values that include hedonic and sensory qualities, but also esthetic, moral, and social qualities (Sirgy, Uysal, & Kruger, 2017). The selection of a particular leisure activity is, thus, a consequence of individual evaluations of expected experiences that are supposed to come along with a leisure activity.

Typically, sport aligns with a plethora of different experiential values. For instance, athletes may seek excitement in adventure sports, social connectedness in team sports, or esthetic forms of self-expression in contemporary dance. In addition, the social context and the organizational setting also matter. Hill and Green (2012) show that customer retention is associated with contextual factors, such as socializing opportunities. Other accounts add more features that shape experiences, e.g., modern equipment, friendly instructors, or harmonious interactions with other members (Min & Breuer, 2018; Papadimitriou & Karteroliotis, 2000; Polyakova & Ramchandani, 2020; Yoshida, 2017). Hence, in addition to the activity itself, the experiential value of leisure sports is also shaped by the social and material environment. This corresponds with the concept of value co-creation, which claims that providers and consumers of sport shape consumption experiences and, thus, create value in a collaborative, interactive process (Horbel, Popp, Woratschek, & Wilson, 2016; Stegmann, Nagel, & Ströbel, 2021; Vargo & Lusch, 2004).

Given that DSE and OSE activities differ in many aspects, it seems likely that typical consumer experiences differ as well. Although initial choices to try out a DSE activity were enforced by the pandemic, any repeated and continuous participation is then based on concrete experiences. Value-based models of consumer choice (Sheth, Newman, & Gross, 1991) suggest that users compare experiential values of DSE offers with their previous experiences of OSE courses and base their decision regarding the continuation of any of these activities on this evaluation. In this regard, Sweeney and Soutar (2001) proposed a framework with multiple dimensions, including a social, emotional, functional, and value-for-money dimension. Hence, only individuals who value their experience in DSE activities on some of these dimensions as (more) positively as their experiences in OSE activities are likely to become regular users. However, there are hardly any accounts that compare experiences of DSE and OSE activities.

This paper aims to add to the understanding of participant experiences in DSE activities by comparing these experiences with experiences in *similar* OSE activities. Two research questions are addressed: (1) Do active participants experience digitally supported sports and exercises (DSE) activities differently compared to offline sports and exercises (OSE) in clubs and gyms? (2) Are there any individual characteristics, such as age or sporting competence, associated with a better evaluation of DSE experiences in relation to OSE experiences? The joint evaluation of DSE and OSE activities will help to assess relative strengths and weaknesses of these offers and allow for tentative conclusions on the future role of DSE after the COVID-19 pandemic.

## Materials and methods

### Study design

The present study is based on a cross-sectional survey, representing physically active adults. The survey was distributed by two large multisport centers in Germany with approximately 15,000 members. Both athletic centers are affiliated with universities, have predominantly student memberships, and offer a full range of sports and fitness activities outside of the lockdown. Active members were invited to participate in an online survey. ‘Active members’ here refers to those members who attended either the gym (and registered at the check-in) or booked a digital course within the 6 months prior to the survey. Overall, 7350 members were contacted by email. Participation in the survey was voluntary and anonymous. The questionnaire was designed to be answered in 15 min. The invitation email contained all necessary information about the survey, content, objectives as well as the research group entrusted with the data analysis, so that individuals could make an informed decision about their participation. Overall, 745 individuals completed the survey, resulting in a response rate of 10%. Data collection took place in March 2021, thus, in the fifth month of the second lockdown period in Germany.

### Sample description

The resulting sample has a mean (*M*) age of 28.8 years (standard deviation [SD] = 10.7). It includes 74% females and 26% males. Given that the group of ‘active users’ is composed of 58% females and 42% males, females are overrepresented. On average, respondents report a good health condition (*M* = 7.18). A majority of 447 respondents (60%) indicated having tried out DSE activities during the 6 months prior to the survey, while 298 respondents (40%) were only engaged in OSE activities. Table 1 shows sociodemographic differences between the DSE and OSE groups.

**Table 1:** Sample description and differences between users of DSE and OSE offers

	Overall (*N* = 745)	OSE only (*N* = 298)	DSE (*N* = 447)	*p*-value
Gender: female	74%	60%	83%	< 0.01
Age^a^	28.77 (10.7)	29.37 (11.6)	28.37 (10.1)	0.86
Self-rated health^a^	7.18 (1.8)	6.76 (1.9)	7.45 (1.6)	< 0.01
Sporting experience^a^	3.66 (2.1)	3.89 (2.1)	3.51 (2.1)	0.01

*OSE* offline sport and exercise activity, *DSE* digital sport and exercise activity

^a^Indicated are means and standard deviations (in brackets)

### Measures

#### Digital sport and exercise activities.

DSE activities are conceptualized as all sport and exercise activities that are essentially supported by or make use of digital media. Participants first indicated whether they engaged in DSE during the lockdown and, if so, additionally indicated which services they have used (*livestreams *or* on-demand videos*). In case they used livestreams, respondents further indicated whether or not they were able to a) see other participants on screen, b) communicate with others via chat functions, c) ask the instructor questions, and d) receive exercise-related corrections from the instructor. We constructed a new variable for *interactivity* based on the sum score of these items.

#### Experiential quality of sport and exercise activities.

A 24-item measure was used to capture six experiential qualities of sport and exercise practices. These experiential qualities relate to affective, social, physical, and motivational experiences, to autonomy and competence. Participants reported their approval of 24 statements on a 4-point Likert scale. To evaluate potential differences between DSE and OSE activities, each statement had to be answered twice, once for DSE and subsequently again for comparable OSE activities. The items and a Principal Component Analysis are fully described in the online supplement.

#### Movement and health competence.

The Physical Activity-Related Health Competence Questionnaire (PAHCO, Sudeck & Pfeifer, 2016; Carl, Sudeck, & Pfeifer, 2020) was used to measure key dimensions of motor and health competence. The subscales used here address mood regulation related to physical activity (PA) (*mood regulation*), control competence for physical training load (*training load*) and physical activity-specific self-control (*self-control*).

#### Sporting experience.

The respondent’s previous sports experience was assessed with a question that referred to the amount of sport practiced during their youth. Answer categories ranged from 0 = “did not engage in sports” to 7 = “more than 7 h/week”.

#### Self-rated health.

Self-rated health was measured with the item “How do you rate your current health”, which could be answered on a 10-point scale from 1 = “very poor” to 10 = “excellent”.

The analyses further include *age* (in years), *gender* and *space at home* (in m^2^). These variables were associated with DSE activity in a previous study (Mutz et al., 2021).

### Analytical approach

After identifying the key dimensions of experiential qualities (see online supplement), we calculated mean scores for each experiential dimension for DSE and OSE activities. Based on all respondents who gave valid answers for DSE and OSE activities, we then compare these means using t‑tests for paired samples. This allows us to assess whether DSE and OSE are perceived differently, in general. In addition, we compute six multiple, ordinary-least-squares (OLS) regression models, one for each experiential dimension. As dependent variables, we use individual difference scores (DS) of each experiential quality (eq), i.e., differences between ratings of DSE vs. similar OSE courses (${DS_{eq1{,}2{,}\ldots }}=\mathrm{DSE}_{eq1{,}2{,}\ldots }-\mathrm{OSE}_{eq1{,}2{,}..}$). Difference scores are meaningful given that consumer choices are often conceptualized as the result of comparisons between different products and their respective qualities (Sweeney & Soutar, 2001). The models include participant characteristics (i.e., the PAHCO subscales, sporting experience, self-rated health, age, gender, space at home), and product characteristics (livestream vs. on-demand, interactivity index). We report unstandardized regression coefficients (*b*) and their significance.

## Results

### Comparison of DSE and OSE experiences

Mean comparisons between DSE and similar OSE activities (Table 2) show that participants evaluate OSE courses as more positive compared to DSE courses on five of six dimensions. The largest difference is found for the *social dimension*: OSE courses have a higher social value for practitioners compared to DSE courses (*M*_DSE_ = 1.75; *M*_OSE_ = 3.39; *p* < 0.01). Participants also evaluate the *physical experience* of DSE and OSE courses differently with OSE being more physically intense compared to DSE (*M*_DSE_ = 2.60; *M*_OSE_ = 3.34; *p* < 0.01). Regarding the *affective quality*, OSE courses are judged as more positive compared to DSE (*M*_DSE_ = 3.16; *M*_OSE_ = 3.71; *p* < 0.01). Moreover, OSE courses have a different *motivational quality* compared to DSE offers: Participants report more inner resistances and less intrinsic motivation to engage in DSE (*M*_DSE_ = 2.56; *M*_OSE_ = 3.14; *p* < 0.01). The difference in the *competence dimension* is relatively small, but also points to an advantage of OSE over DSE (*M*_DSE_ = 2.66; *M*_OSE_ = 2.89; *p* < 0.01). The only advantage of DSE relates to the *autonomy experience*, where DSE courses are evaluated to be better compared to OSE courses (*M*_DSE_ = 3.03; *M*_OSE_ = 2.70; *p* < 0.01).

**Table 2:** Mean differences in experiential values between digital sport and exercise activities and similar offline, on-site activities

Experiential dimension	DSE		OSE			
	*M*	*SD*	*M*	*SD*	*Diff*	*d*
(1) Affective dimension	3.16	0.54	3.71	0.41	0.55**	0.88
(2) Social dimension	1.75	0.76	3.39	0.63	1.64**	1.75
(3) Physical dimension	2.60	0.63	3.34	0.49	0.75**	1.03
(4) Autonomy dimension	3.03	0.61	2.70	0.57	−0.33**	−0.41
(5) Competence dimension	2.66	0.60	2.89	0.53	0.23**	0.38
(6) Motivational dimension	2.56	0.71	3.14	0.61	0.58**	0.69

Paired samples t‑tests

*OSE* offline sport and exercise activity, *DSE* digital sport and exercise activity, *M* mean, *SD* standard deviation

Significance: *p < 0.05, **p < 0.01

### Predictors of individual experiences of DSE compared to OSE courses

Multiple regression models reveal that some individual characteristics are associated with the evaluation of DSE courses in relation to similar OSE activities (Table 3).

**Table 3:** Regression models for perceived differences between DSE and OSE courses

	Affective dimension	Social dimension	Physical dimension	Autonomy dimension	Competence dimension	Motivational dimension
Intercept	−0.80	−2.06	−1.09	0.25	−0.86	−1.37
**Product variables**						
Livestream	0.08	0.04	0.12	−0.20*	0.10	0.29**
Interactivity	−0.02	0.19**	−0.00	−0.11**	−0.06*	−0.04
**Participant variables**						
PAHCO mood regulation	−0.17**	−0.16*	−0.17*	−0.16*	−0.22**	−0.21**
PAHCO training load	−0.06	0.01	−0.03	−0.05	0.21**	0.02
PAHCO self-control	0.14**	0.10**	0.18**	0.05	0.16**	0.27**
Sporting experience	−0.02	−0.03	0.01	−0.02	−0.01	−0.05**
Self-rated health	0.07**	0.08**	0.04	0.05*	0.04*	0.06*
Control variables						
Age (in years)	0.00	0.00	0.00	0.01	0.00	0.00
Gender (female)	0.07	0.11	0.04	0.29**	0.10	0.15
Space at home (in m^2^)	0.00	−0.07	0.03	0.02	−0.04	0.02
**Model fit (R**^**2**^**)**	0.085	0.158	0.058	0.124	0.134	0.138

Linear regression models

*OSE* offline sport and exercise activity, *DSE* digital sport and exercise activity, *PAHCO* Physical Activity-Related Health Competence Questionnaire

Dependent variables are individual difference scores (DSE **−** OSE), where *positive values* indicate an advantage and *negative values* a disadvantage of DSE compared to OSE. Table shows unstandardized regression coefficients. Significance: *p < 0.05, **p < 0.01

Results for the *affective dimension* of DSE reveal that PA-related self-control (*b* = 0.14, *p* < 0.01) and subjective health (*b* = 0.07, *p* < 0.01) are associated with a better judgement of the affective value of DSE compared to OSE. Higher scores in PA-related mood regulation (*b* = −0.17, *p* < 0.01) are associated with a more critical evaluation of DSE.

The *social quality* of DSE is evaluated more positively by users of more interactive DSE courses (*b* = 0.19, *p* < 0.01) as well as individuals with better sports-related self-control (*b* = 0.10, *p* < 0.01) and better subjective health (*b* = 0.08, *p* < 0.01). A higher competence in PA-related mood regulation is negatively related to social quality judgements (*b* = −0.16, *p* < 0.05).

Quite similar results are shown for the *physical quality*: A better PA-related self-control (*b* = 0.18, *p* < 0.01) correlates with a better evaluation of the physical dimension of DSE. Higher scores in PA-related mood regulation is again associated with a more critical evaluation of the physical quality of DSE compared to OSE (*b* = −0.17, *p* < 0.01).

With regard to *autonomy*, the model reveals that livestreamed DSE is evaluated more critically than on-demand videos (*b* = −0.20, *p* < 0.05). More interactivity also comes at the cost of autonomy experiences (*b* = −0.11, *p* < 0.01). Autonomy is judged significantly higher by females (*b* = 0.29, *p* < 0.01) and healthier individuals (*b* = 0.05, *p* < 0.05). Respondents with a higher competence in PA-related mood regulation rate the autonomy quality of DSE more critically (*b* = −0.16, *p* < 0.05).

The *competence dimension* of DSE is rated lower, when DSE is more interactive (*b* = −0.06, *p* < 0.05). PA-related training competence (*b* = 0.21, *p* < 0.01), self-control (*b* = 0.16, *p* < 0.01), and self-rated health (*b* = 0.04, *p* < 0.05) predict a higher competence perception in DSE compared to OSE. PA-related mood regulation again aligns with a more skeptical judgement (*b* = −0.22, *p* < 0.01).

The *motivational quality* of DSE is assessed better by participants of live streamed DSE programs (*b* = 0.29, *p* < 0.01). PA-related self-control (*b* = 0.27, *p* < 0.01) and subjective health (*b* = 0.06, *p* < 0.05) are both associated with higher motivational values of DSE. A higher competence in PA-related mood regulation (*b* = −0.21, *p* < 0.01) and more sporting experience in youth (*b* = −0.05, *p* < 0.01) are associated with a more negative evaluation of the motivational quality.

## Discussion

This brief report compared experiences of participants in DSE activities with their experiences in similar OSE activities, thereby revealing specific strengths and weaknesses. Following the idea that consumption experiences are multidimensional (e.g., Polyakova & Ramchandani, 2020), we distinguished affective, social, physical, autonomy, competence and motivational qualities. Findings show that DSE is associated with a higher level of autonomy than OSE. Consumer autonomy comes from the time-independent use of DSE, the variety of videos to choose from and the freedom to adapt or omit some of the exercises shown. However, in all other dimensions DSE is perceived as less positive as OSE: It has a lower affective value, is rated physically less demanding, users feel less competent when exercising, and report lower intrinsic motivation. Most notably, however, they judge the social aspect of DSE less positive.

Regression models indicate that the format of DSE offers matters: Live-streamed DSE courses have a higher motivational value than recorded videos. However, this comes at the cost of autonomy as livestreams reduce the independence of users regarding the time and type of exercises. Digital features that allow for interactions (e.g., chat functions) help to add to the social value of DSE, but are negatively associated with the feeling of competence. This trade-off may result from the fact that communicating during live-streamed workout is limited by technology and usually requires an interruption of the exercise (Gui, Tsai, Vajda, & Carroll, 2022).

With regard to individual characteristics, results show that participants with higher PA-related self-control scores judge DSE activities better. The effect of self-regulation is plausible given that the lack of fixed schedules and routines in DSE requires more self-regulation and self-discipline. Participants with a better health status also judge DSE activities somewhat better than users with health issues. It can be conjectured that a good health means that participants can choose from a large variety of (on-demand) DSE courses, whereas users with lower self-rated health may be more insecure to choose appropriate activities that fit their physical ability. All models also revealed a more negative evaluation of DSE compared to OSE activities from participants with a higher PA-related mood regulation competence. Social psychologists argue that emotional episodes are embedded in social interactions and stress that emotional contagion is an interpersonal process (Friesen et al., 2013). It can be assumed, however, that athletes with a higher competence in mood regulation are less attracted by DSE, because the standardized training in an isolated environment is less suitable for mood regulation.

Sport clubs capacities to prioritize digitalization are often limited (Ehnold, Steinbach, & Schlesinger, 2020). To make effective decisions with regard to DSE, they need to know how consumers judge DSE in relation to alternative offers. Our findings suggest that a majority of DSE participants will prefer OSE over DSE, when choice is not restricted by the pandemic. Only for individuals who put high value to autonomy may DSE permanently become the first choice. From a management perspective, it is worth noting that the production of livestreams requires similar resources but usually reaches a smaller audience compared to on-demand videos, so that livestreams should offer added value for customers that justifies the higher relative production costs. Findings, however, do not indicate that this effort is worthwhile, as exercising in live workouts is generally not experienced better as the use of on-demand videos.

The lack of social interactions is the largest shortcoming of DSE. It would be advisable to link DSE more closely to social communities, include interactive modules, or performance-based challenges to increase the social value of DSE activities for users (Gui et al., 2022). These features would allow users to become more active collaborators, adding utility or meaning to the sports offering, which is in accordance with the idea that value is co-created by consumers (Vargo & Lusch, 2004). In addition, technical options for individualized feedback or individual choices regarding music selection or trainer instruction could also improve the evaluation of DSE. Being able to make flexible and individualized adaptation within programs could become more important in the future. In addition, DSE can become a regular option for individuals and social groups with limited time or restricted mobility, which is already widely discussed in the current debates around eHealth (Tebeje & Klein, 2021).

Besides its strengths, this study also has limitations: The low average age of our sample limits conclusions about older age-groups and their typical experience of DSE. The DSE activities researched in this study are predominantly fitness-oriented. In this respect, it is questionable to what extent results are also valid for team sports or other types of sport that include more interactions. It will be the task of further studies to investigate differences between various forms of DSE offerings that are currently becoming highly differentiated as well as to expand the scope of the analysis to different age groups. The low response rate of the survey limits generalizability and makes it more likely that selection bias may exist. In this regard, it is noticeable that women are overrepresented in the sample. Although more women engage in DSE courses than men (Mutz et al., 2021), the high share of female respondents still raises the question of whether the survey topic was of less interest to men. Regarding the measurement of self-rated health, we are aware that validated scales exist. However, single item measures are often preferred in surveys, like here, to reduce questionnaire length and avoid break-off. Finally, due to the cross-sectional design, findings can only represent one phase of the pandemic. As society moves into a stage where the coronavirus is endemic, it will be necessary to continue to monitor the long-term impact of the pandemic for the sporting landscape and the role of DSE.

## Supplementary Information

The online supplement documents the results of a Principal Components Analysis (PCA) with the 24 items that we considered for assessing experiential qualities of digital sport and exercise activities

## Declarations

All participants provided written informed consent prior to completing the survey. The data have been processed in accordance with the General Data Protection Regulations.
