# Supplementary material for: Just a stopgap for ‘real’ sports? Experiences with digital sport and exercise activities during the COVID-19 pandemic
Source: Ger J Exerc Sport Res. 2023 Apr 25:1–6. Online ahead of print. doi: 10.1007/s12662-023-00881-y (PMC10129302; doi:10.1007/s12662-023-00881-y)
Supplement: Supplementary file 1 — The online supplement documents the results of a Principal Components Analysis (PCA) with the 24 items that we considered for assessing experiential qualities of digital sport and exercise activities [file 12662_2023_881_MOESM1_ESM.docx]

Just a Stopgap for ‘Real’ Sports? Consumer Experiences with Digital Sport and Exercise Activities during the COVID-19 Pandemic

**Online Supplement**

We conducted a Principal Components Analysis (PCA) with the 24 items that we considered for assessing experiential qualities of digital sport and exercise (DSE) activities. PCA is a method that aims to reduce complex data structures to a few principal components. In an iterative procedure, several principal components are identified, each of which accounts for the largest share of the (remaining) variance. In order to actually identify only the most important components and not any number of components, we resort to the Kaiser criterion, according to which factors with Eigenvalues >1 are not taken into account. Moreover, as the experiential values we seek to identify likely correlate with each other, we refrain from the orthogonality assumption and use the oblique transformation instead (*oblimin*).

The PCA suggests a solution with six factors that account for 64% of the total variance. The six factors with Eigenvalues >1.0 refer to the affective, social, physical, autonomy, competence and motivational experience of DSE activities (Table A1). Although the oblimin procedure allows for correlations between the principal components, all correlations are <.30.

1) The first factor captures the “*affective experience*”, i.e. the mood and emotions experienced when doing sports and exercising.

2) The second factor refers to the “*social experience*”, that is, the feeling of being connected to others and being part of a group.

3) The third component is labelled “*physical experience*” and refers to the intensity and the degree of exhaustion related to sport and exercise.

4) The fourth factor refers to “*autonomy*” and combines experiences of independence, choice and freedom to adapt exercises according to one’s own needs.

5) The fifth component relates to “*competence*”, i.e. the confidence to be able to perform all exercises correctly.

6) Finally, a sixth factor captures the “*motivational experience*”, here understood as intrinsic motivation that allows to do sport and exercises without inner resistance or pressures.

Following the recommendations by Wolff and Bacher (2010), we specify that a variable should load at least with +/-.50 on one factor and not on any other factor higher than +/-.30. This way, 22 of the 24 variables can be clearly assigned to one factor. These variables were then used to calculate the scales, reported in our article. Two items (19 and 20), however, are excluded because they do not load on one factor, according to the specifications above.

**References**

Wolff, H.G. & Bacher, J. (2010). Hauptkomponentenanalyse und explorative Faktorenanalyse. In: C. Wolf & H. Best (eds), *Handbuch der sozialwissenschaftlichen Datenanalyse* (pp. 333-365). Wiesbaden: VS Verlag für Sozialwissenschaften.

***Table A1****: Dimensions of user experiences with digital sport and exercise activities*

|  | **Principal Components** | | | | | |
| --- | --- | --- | --- | --- | --- | --- |
|  | I | II | III | IV | V | VI |
| **Affective dimension (24.9%, EV=5.98)** |  |  |  |  |  |  |
| 1. I feel very well when exercising. | **.60** | -.05 | .06 | .19 | .10 | .14 |
| 1. I am able to forget daily hassles when exercising. | **.72** | -.17 | .08 | .04 | -.06 | .14 |
| 1. I am in a good mood afterwards. | **.75** | -.06 | .09 | .06 | .03 | .06 |
| 1. It brings a pleasant stimulation to my life. | **.71** | -.04 | .07 | .04 | -.04 | .13 |
| **Social dimension (13.0%, EV=3.11)** |  |  |  |  |  |  |
| 1. I am able to get in contact with others. | .03 | **-.74** | -.01 | -.02 | -.10 | .04 |
| 1. I feel as a part of a group. | .14 | **-.84** | .05 | -.19 | .07 | .00 |
| 1. I feel a team spirit when exercising. | .13 | **-.84** | .07 | -.14 | .03 | -.01 |
| 1. I feel connected to other people. | .09 | **-.85** | .04 | -.09 | .07 | -.02 |
| **Physical dimension (8.8%, EV=2.10)** |  |  |  |  |  |  |
| 1. I often push myself to my limits. | .12 | -.08 | **.77** | -.04 | .04 | -.01 |
| 1. It am often sweaty and strenuous when exercising. | .11 | -.05 | **.82** | .06 | -.08 | -.03 |
| 1. I rarely push myself so hard that I am out of breath.* | -.12 | .00 | **.72** | -.07 | .12 | -.07 |
| 1. I am able to completely exhaust myself. | .10 | .03 | **.79** | .10 | .01 | .07 |
| **Autonomy dimension (7.0%, EV=1.67)** |  |  |  |  |  |  |
| 1. I can choose from a great variety of offers. | -.00 | .22 | .17 | **.63** | -.10 | .08 |
| 1. I am able to exercise when and where I want. | .09 | .19 | .04 | **.71** | -.12 | .09 |
| 1. I feel free to adapt exercises the way I want. | .22 | -.09 | -.13 | **.58** | .19 | -.18 |
| 1. I am able to exercise independently. | .14 | .09 | .00 | **.70** | .14 | -.19 |
| **Competency dimension (5.7%, EV=1.37)** |  |  |  |  |  |  |
| (17) I always feel competent when exercising. | .02 | .01 | .07 | .05 | **.77** | .18 |
| (18) I am often insecure if I do it right.* | -.02 | .03 | .11 | .01 | **.84** | .09 |
| (19) I can learn a lot from watching others.*^+^ | -.26 | -.50 | .10 | .34 | -.28 | .18 |
| (20) I always know how to perform movements correctly^+^ | -.17 | -.33 | -.04 | .39 | .26 | .08 |
| **Motivational dimension (4.9%, EV=1.17)** |  |  |  |  |  |  |
| (21) I have to get over myself to exercise.* | .11 | -.02 | -.05 | .01 | .03 | **.84** |
| (22) I often feel some inner resistance before exercising.* | -.03 | -.04 | .01 | -.10 | .17 | **.79** |
| (23) I exercise more out of a sense of duty.* | .03 | .02 | -.03 | -.02 | .04 | **.84** |
| (24) I am always looking forward to exercise. | .25 | .03 | .04 | -.01 | .01 | **.70** |

Notes: Principal Components Analysis with Oblimin rotation. *Reverted item; +Item deleted from the scale.
